# Engineered NS1 for Sensitive, Specific Zika Virus Diagnosis from Patient Serology

**DOI:** 10.3201/eid2705.190121

**Published:** 2021-05

**Authors:** Thai Leong Yap, Shin Yee Hong, Jun Hui Soh, Lekha Ravichandraprabhu, Vanessa W.X. Lim, Hsi-Min Chan, Tommy Z.X. Ong, Ying Ping Chua, Shi En Koh, Huajing Wang, Yee Sin Leo, Jackie Y. Ying, William Sun

**Affiliations:** Experimental Drug Development Centre, Singapore (T.L. Yap, S.Y. Hong);; Institute of Bioengineering and Nanotechnology, Singapore (T.L. Yap, S.Y. Hong, L. Ravichandraprabhu, T.Z.X. Ong, Y.P. Chua, S.E. Koh, H. Wang, W. Sun);; NanoBio Lab, Singapore (J.H. Soh, H.-M. Chan, J.Y. Ying);; National Centre for Infectious Diseases, Singapore (V.W.X. Lim, Y.S. Leo);; Tan Tock Seng Hospital, Singapore (Y.S. Leo);; Yong Loo Lin School of Medicine, National University of Singapore (Y.S. Leo, W. Sun).

**Keywords:** Zika, dengue, flavivirus, viruses, monoclonal antibody, serologic test, microcephaly, immunochromatography, diagnostics, immunoglobulin, enzyme-linked immunosorbent assay, Zika virus

## Abstract

Dengue virus (DENV) and Zika virus (ZIKV) belong to the *Flaviviridae* family of viruses spread by A*edes aegypti* mosquitoes in tropical and subtropical areas. Accurate diagnostic tests to differentiate the 2 infections are necessary for patient management and disease control. Using characterized ZIKV and DENV patient plasma in a blind manner, we validated an ELISA and a rapid immunochromatographic test for ZIKV detection. We engineered the ZIKV nonstructural protein 1 (NS1) for sensitive serologic detection with low cross reactivity against dengue and developed monoclonal antibodies specific for the ZIKV NS1 antigen. As expected, the serologic assays performed better with convalescent than acute plasma samples; the sensitivity ranged from 71% to 88%, depending on the performance of individual tests (IgM/IgG/NS1). Although serologic tests were generally less sensitive with acute samples, our ZIKV NS1 antibodies were able to complement the serologic tests to achieve greater sensitivity for detecting early infections.

Zika virus (ZIKV), a single-stranded RNA virus, belongs to the family *Flaviviridae*. It is transmitted by infected *Aedes* spp. mosquitoes, the same vector that transmits dengue virus (DENV) in tropical and subtropical areas (*1**–**3*). Patients infected by ZIKV are often asymptomatic or have mild symptoms similar to those of dengue infections, such as fever, rash, and joint pain (*4**–**6*). However, the ZIKV outbreak in Brazil in 2015–2016 has drawn much attention because of its association with a marked increase in the number of newborns with microcephaly from infected mothers (*7**–**10*). Other neurologic diseases, such as Guillain-Barré syndrome, have also been associated with ZIKV infections (*7*,*11*,*12*).

Several molecular- or serologic-based assays have been approved by the US Food and Drug Administration for emergency use to diagnose ZIKV infections (*13*,*14*). Nucleic acid testing has shown good specificity in general, but high variations in assay sensitivity have been reported (*15*). This variability can be the result of complicated experimental setups, genetic variability in different virus strains, or narrow detection window because of low viremia load in ZIKV-infected patients (*16*,*17*). Thus, in nucleic acid test–negative cases, complementary assays based on serology testing, such as Zika IgM antibody capture ELISA (MAC-ELISA) and plaque-reduction neutralization test (PRNT), are required to validate the results (*18*,*19*). Those secondary tests are not specific because of high cross reactivity with other flaviviruses, further complicating the interpretation of test results (*20*,*21*). There is a need to develop a more reliable Zika diagnostic test for outbreak control and improved patient care.

We aimed to develop specific serology tests that could differentiate ZIKV from DENV infections by engineering the ZIKV nonstructural protein 1 (NS1). We established both ELISA and immunochromatographic assays (IAs) for specific and sensitive binding to ZIKV IgM and IgG. In particular, we developed 2 IA assays, in which the engineered antigens were used either as capture (F1 format) or detector (F2 format), resulting in slight difference in sensitivity and specificity. We further assessed assay performance by testing plasma samples collected from patients during acute and convalescent phases of infection. 

## Materials and Methods

### Patient Samples and Study Approval

Whole-blood samples were collected with ethylenediaminetetraacetic acid-lined Vacutainer tubes (Becton Dickinson, http://www.bd.com) from patients referred to the Communicable Disease Centre, Tan Tock Seng Hospital (TTSH), Singapore. We obtained blood specimens from patients consenting to the study. All patients gave separate written informed consent. The study protocols were approved by the SingHealth Centralized Institutional Review Board (reference no. 2016/2219) and by the National Healthcare Group Domain Specific Review Board (reference no. 2015/00528).

This study included plasma samples obtained from 94 patients with ZIKV who were admitted to the Communicable Disease Centre at TTSH during August 27, 2016–August 14, 2017, and 70 DENV patients admitted during April 29, 2016–March 28, 2017. Samples were collected at 2 phases: acute (1–6 days postonset of symptoms [dpo]) and early convalescent (7–21 dpo). Patients could donate blood samples multiple times during each phase. Only 11/94 (12%) of patients from the ZIKV cohort and 12/70 (17%) of patients from the DENV cohort had traveled within 2 weeks of recruitment. Therefore, we could conclude that most patients were infected from local transmission.

Among the patients with ZIKV, 41 (43.62%) were female and 53 (56.38%) were male (Table 1). These patients were confirmed to be infected with ZIKV according to reverse transcription PCR (RT-PCR) using an adapted protocol (*22*) performed on plasma and urine samples obtained during their first visits. In addition, all ZIKV patients were tested for dengue NS1 using the SD BIOLINE Dengue Duo rapid test (Abbott, https://www.globalpointofcare.abbott); 3 of 94 patients were further confirmed DENV NS1-positive by RT-PCR, indicating a concurrent DENV infection (*23*). Among the DENV patients, 19 (27.14%) were female and 51 (72.86%) were male. The patients with DENV were tested with hospital routine diagnostics using the SD BIOLINE Dengue Duo rapid test. All NS1-positive samples were confirmed to be dengue positive using the FTD Zika/dengue/chikungunya RT-PCR (Fast Track Diagnostics, http://www.fast-trackdiagnostics.com). Dengue serotypes were further determined by FTD dengue differentiation RT-PCR test (Fast Track Diagnostics), according to the manufacturer’s instructions (Appendix 1).

**Table 1:** Characteristics of patients admitted to Tan Tock Seng Hospital, Singapore, whose blood samples were used for study of Zika diagnosis*

Patient characteristics	Patients with Zika virus	Patients with dengue virus
Total no.	94	70
Sex		
M	53 (56.4)	51 (72.9)
F	41 (43.6)	19 (27.1)
Ethnicity		
Chinese	77 (81.9)	41 (58.6)
Malay	7 (7.4)	5 (7.1)
Indian	5 (5.3)	7 (10.0)
Other	5 (5.3)	17 (24.3)
Median age, y	39	35
Age range, y	14–72	22–60

*Values are no. (%) except as indicated.

For the validation tests, we used 70 samples from 62 unique patients with ZIKV (9 patients had >1 sample collected during the time period), and 81 samples from 68 unique patients with DENV (13 patients had >1 sample collected) collected in the acute phase (1–6 dpo). From the early convalescent phase (7–21 dpo), we used 48 samples from 44 unique patients with ZIKV and 70 samples from 53 unique patients with DENV. Samples were randomized and blinded during testing.

During assay optimization, we used a subset of samples from TTSH and a commercial vendor (SeraCare, https://www.seracare.com) and designated this combined sample pool as the training set (37 ZIKV samples, 67 DENV samples). TTSH samples have records of the day of collection after onset of symptoms (27 ZIKV samples, 46 DENV samples), whereas this information was not available for the commercial samples (10 ZIKV samples, 21 DENV samples). SeraCare panels 0845–0142 (ZIKV) and 0845–0074 (DENV) were used for training; samples DSC-7, 12, and 20 from SeraCare panel 0845–0051 (DENV) and ZPC-1, -2, -4, and -8 (ZIKV, country of origin Columbia) acquired from Precision Technologies, Singapore (http://www.pretech.com.sg) were used for characterization of engineered ZIKV NS1 (Appendix 1). 

## Results

### Engineering Full-Length NS1 Protein for Serologic Assays

We hypothesized that ZIKV NS1 could be used to develop a specific and sensitive serologic test because we were able to generate monoclonal antibodies specific for this antigen without cross-reactivity to NS1 from other flaviviruses. When we first tried to express the full-length ZIKV NS1 protein (GenBank accession no. KX447521.1), we found that it was poorly expressed in our mammalian system. We subsequently constructed various ZIKV NS1 domains fused to different carriers at the N or C terminus. We aimed to optimize the construct with respect to solubility and specific reactivity to ZIKV immune serum samples.

**Figure 1:**
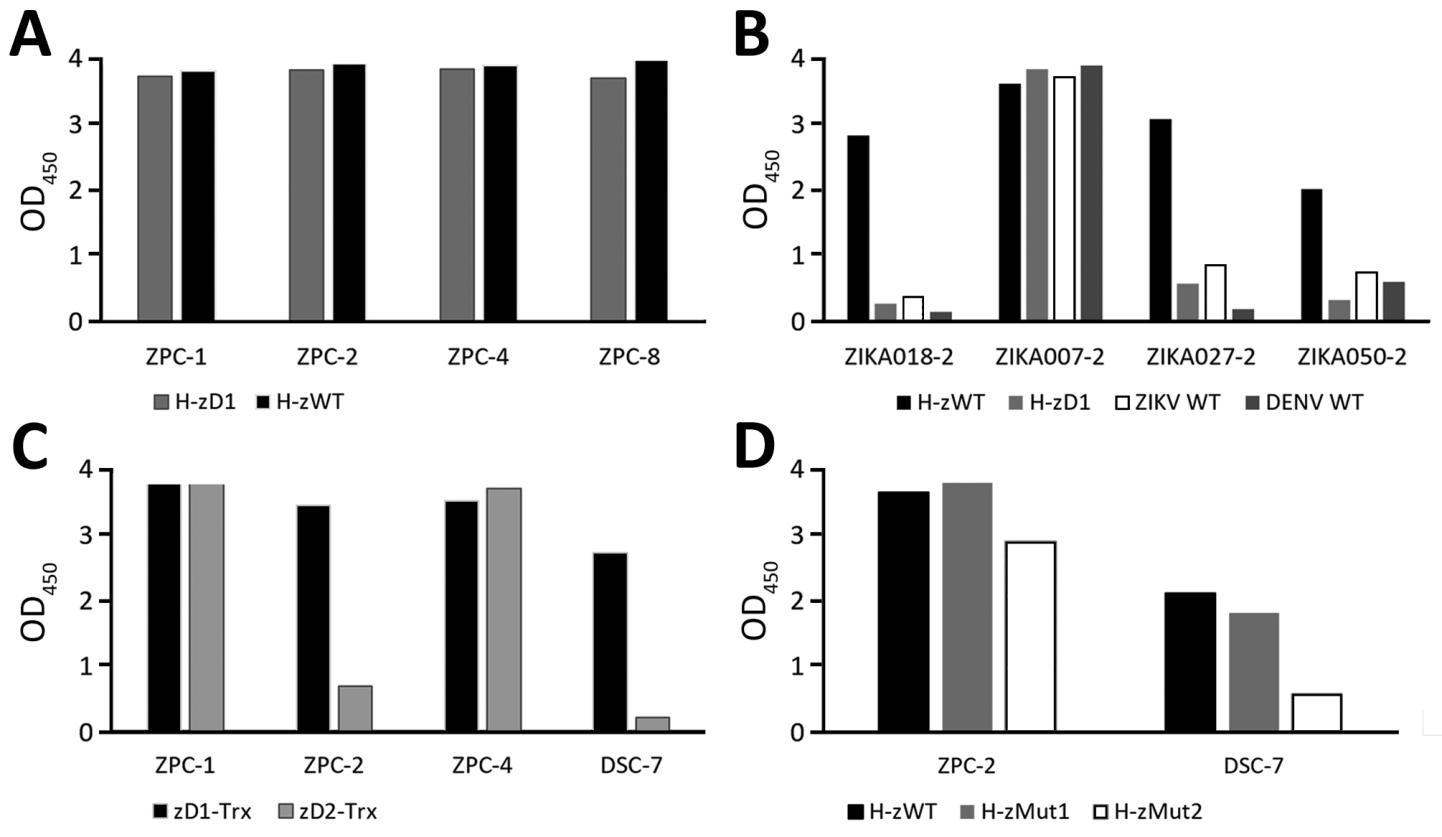
Reactivity of nonstructural protein 1 antigens to ZIKV and DENV plasma in study of Zika diagnosis, Singapore. A) Reactivity of H-zWT and H-zD1 to commercial ZIKV IgG in ELISA format. B) Reactivity of H-zWT, H-zD1, ZIKV WT, and DENV WT to samples from Tan Tock Seng Hospital. C) Comparison of zD1-Trx and zD2-Trx activity to DSC-7. D) comparison of H-zWT, H-zMut1, and H-zMut2 activity to DSC-7. The graphs show mean OD measurements from 2 replicates. DENV, dengue virus; OD, optical density; WT, wild type; ZIKV, Zika virus.

Among the different construct designs, we determined that the His-tagged albumin domain (H, residue 1–197 aa) N terminally fused to the NS1 variants, resulting in H-zWT (NS1 1–352 aa) and H-zD1 (NS1 172–352 aa), showed reasonable solubility (>1 mg per 40–80 mL of culture). Using IgG ELISA, we showed that the 2 constructs had good reactivity to the commercial ZIKV samples (Figure 1, panel A), but H-zD1 showed reactivity to only 1 TTSH ZIKV sample (Figure 1, panel B). We observed that wild-type NS1 (ZIKV WT and DENV WT, obtained from Native Antigen) showed similar reactivity as H-zD1 to these TTSH serum samples (Figure 1, panel B).

Although our full-length ZIKV NS1 was not expressed in soluble form with the thioredoxin (Trx) at the C terminus, we were able to produce 2 soluble forms of C terminal constructs: zD1-Trx (residue 172–352 aa) and zD2-Trx (172–339 aa). We asked whether truncation at the C terminus could differentiate zD1-Trx from zD2-Trx in DENV IgG cross reactivity. Among the DENV samples from the SeraCare commercial panel 0845_0051 that were available at the time (DSC-7, DSC-12, and DSC-20), we found that DSC-7 showed cross reactivity to the ZIKV WT. We then showed that zD2-Trx has reduced IgG ELISA activity to DSC-7, compared with zD1-Trx (Figure 1, panel C). Although we observed this only with 1 DENV serum sample, we hypothesized that, by altering residues conserved between DENV and ZIKV in the region of 339–352 aa, we could reduce DENV IgG cross reactivity.

**Figure 2:**
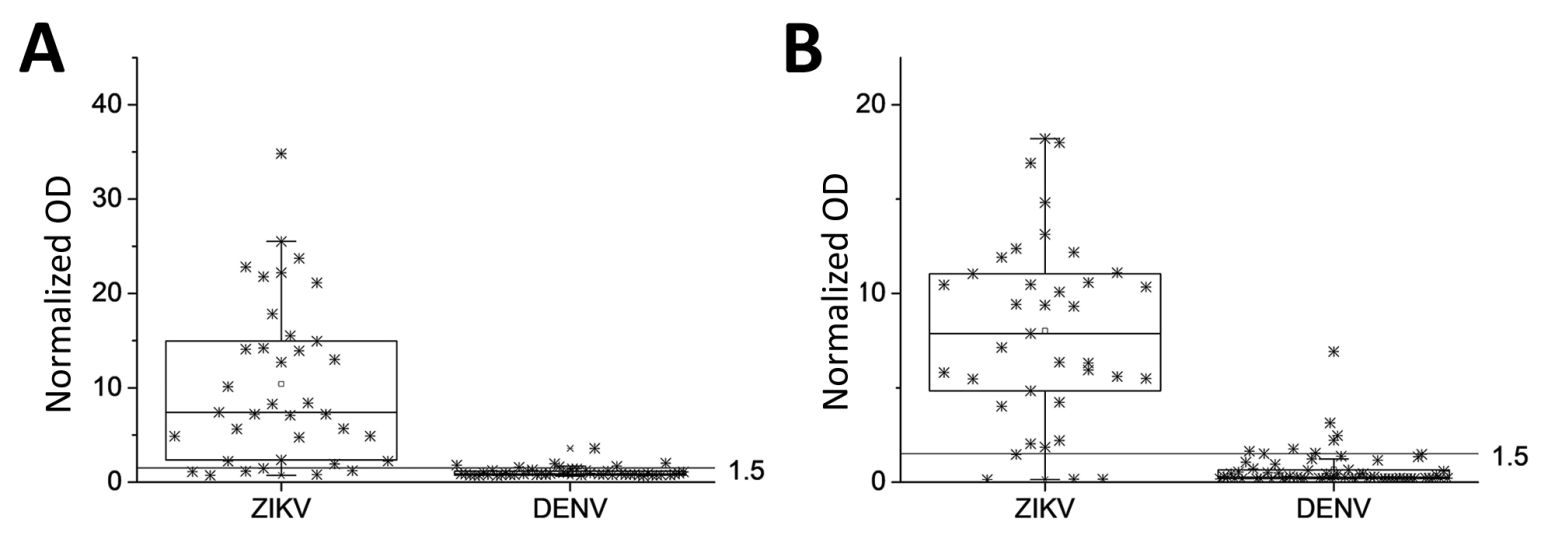
Reactivity of nonstructural protein 1 antigens to ZIKV and DENV plasma in study of Zika diagnosis, Singapore. H-zMut2 ELISA was tested with a training set for binding to IgM (A) and IgG (B). Results are representative of replicates for each sample. Normalized OD >1.5 for plasma or serum sample was determined as positive for ZIKV infection. DENV, dengue virus; OD, optical density; ZIKA, Zika virus.

We subsequently generated a series of mutants spanning the 339–352 aa region of the H-zWT construct because this format was the most reactive to ZIKV IgG. Of all the mutants, we selected H-zMut1 (V350T, N344D, P341Q) and H-zMut2 (A352D, T351H, S348D, N344K, P341H), for their soluble expression and their ability to reduce DENV cross reactivity without greatly compromising the ZIKV signal in both the ELISA and IA formats. We first showed that H-zMut2 had a greater reduction in reactivity to DSC-7 compared with H-zWT and H-zMut1 in IgG ELISA (Figure 1, panel D). We then further used H-zMut2 as the capture antigen for optimizing the ELISA for specific binding to IgM and IgG with a collection of plasma samples designated the “training set.” Under the optimized ELISA conditions, H-zMut2 resulted in IgM/IgG detection with sensitivity and specificity >80% (Figure 2, panels A, B; Appendix 1 Table 1).

### H-zMut2 ELISA for Blinded Test Evaluation

**Figure 3:**
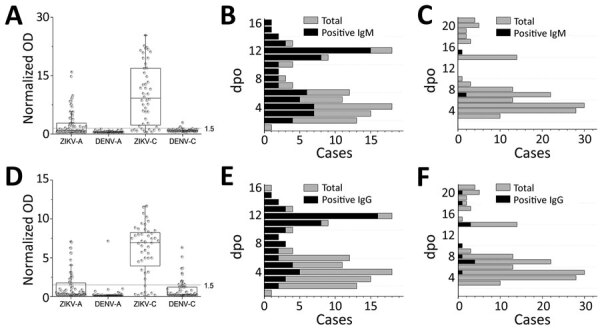
H-zMut2 ELISA for validation set in study of Zika diagnosis, Singapore. A, D) H-zMut2 reactivity to IgM (A) and IgG (D) present in plasma collected during acute and recent convalescent phases (ZIKV-A, n = 70 [1–6 dpo]; ZIKV-C, n = 48 [7–14 dpo]; DENV-A, n = 81 [1–6 dpo]; DENV-B, n = 70 [7–21 dpo]). Plasma samples were blinded and tested with H-zMut2 as the capture antigen. Normalized OD >1.5 for plasma sample was determined as positive for ZIKV infection. Results are representative of 2 replicates for each plasma sample. B, C, E, F) Patient samples for ZIKV (B, E) and DENV (C, F). The plots show distribution of number of plasma cases (*x*-axis) over number of days post infection (*y*-axis, dpo) for H-zMut2 ELISA tested with validation set; the number of positive plasma samples (black bar) was shown against the total (gray bar) for each dpo. DENV, dengue virus; dpo, days postonset of symptoms; OD, optical density; ZIKA, Zika virus.

Upon achieving the desired performance with the training set, we proceeded to evaluate our assay on a larger group of samples in a blinded manner. This validation set consisted of 269 samples collected by TTSH from patients with ZIKV and DENV. Among the 3 engineered antigens, H-zMut2 showed greater detection sensitivity and specificity than ZIKV WT but only slightly lower sensitivity (though higher specificity) compared with H-zWT (Figure 3; Appendix 1 Table 2). In the ELISA test, H-zMut2 showed low sensitivity with acute samples (IgM/IgG 41%/23%) but high specificity (IgM/IgG 100%/97%) (Table 2; Figure 3). The result reflected the low IgG titer during the acute phase of Zika infection, consistent with other studies (Table 2; Figure 3, panels D, E; Appendix 1 Table 2). Compared with H-zMut2, ZIKV WT showed much lower sensitivity (IgM/IgG 3%/14%) (Appendix 1 Table 2). In contrast with the acute samples, H-zMut2 capture antigen showed relatively high sensitivity when tested on convalescent samples (IgM/IgG sensitivity 79%/83%, IgM/IgG specificity 95%/84%) (Table 2; Figure 3), and continued to outperform ZIKV WT (IgM/IgG sensitivity 33%/56%, IgM/IgG specificity 98%/73%) (Appendix 1 Table 2).

**Table 2:** Sensitivity and specificity results for validation set in blinded evalution for study of Zika diagnosis, Singapore*

Phase	Sensitivity, % (95% CI)				Specificity, % (95% CI)		
	ELISA	Lateral flow			ELISA	Lateral flow	
		F1	F2			F1	F2
Acute, 1–6 dpo							
IgM	41.4 (29.8–53.8)	51.4 (39.2–63.6)	50.0 (37.8–62.2)		100.0 (95.5–100.0)	95.1 (87.8–98.6)	97.5 (0.91–1.00)
IgG	22.9 (13.7–34.4)	44.3 (32.4–56.7)	20.0 (11.4–31.3)		98.8 (93.3–100.0)	92.6 (84.6–97.2)	98.8 (0.93–1.00)
IgM/IgG	52.9 (40.6–64.9)	68.6 (56.4–79.1)	60.0 (47.6–71.5)		98.8 (93.3–100.0)	88.9 (80.0–94.8)	96.3 (0.90–0.99)
NS1	41.4 (29.8–53.8)	NP	NP		97.5 (91.4–99.7)	NP	NP
IgM/NS1	55.7 (43.3–67.6)	NP	NP		97.5 (91.4–99.7)	NP	NP
IgG/NS1	61.4 (49.0–72.8)	NP	NP		96.3 (89.6–99.2)	NP	NP
IgM/IgG/NS1	67.1 (54.9–77.9)	NP	NP		96.3 (89.6–99.2)	NP	NP
Convalescent, 7–21 dpo							
IgM	79.2 (65.0–89.5)	70.8 (55.9–83.0)	70.8 (55.9–83.0)		95.7 (88.0–99.1)	87.1 (77.0–93.9)	94.3 (86.0–98.4)
IgG	83.3 (69.8–92.5)	89.6 (77.3–96.5)	79.2 (65.0–89.5)		84.3 (73.6–91.9)	78.6 (67.1–87.5)	90.0 (80.5–95.9)
IgM/IgG	89.6 (77.3–96.5)	89.6 (77.3–96.5)	87.5 (74.8–95.3)		80 (68.7–88.6)	68.6 (56.4–79.1)	84.3 (73.6–91.9)

*ELISA and IA assays were evaluated for the detection of NS1, IgM, and IgG with TTSH plasma samples (ZIKV: n = 70 with 1–6 dpo, and n = 48 with 7–16 dpo; DENV: n = 81 with 1–6 dpo, and n = 70 with 7–21 dpo. Sensitivity and specificity were determined with positive plasmas divided by the total number of respective ZIKV and DENV plasma samples. DENV, dengue virus; dpo, days postonset of symptoms; F1, capture format; F2, detector format; IA, immunochromatographic assay; NP, not performed (NS1 antigen test was not performed in the lateral flow formats because of low sensitivity); NS1, nonstructural protein 1; ZIKV, Zika virus.

Given that the IgM or IgG ELISA with H-zMut2 each detected a different subset of ZIKV samples (Figure 3, panels B, E), combining the IgM/IgG test results could achieve a greater sensitivity for both acute samples (17% [WT] < 52% [mut2]) and convalescent samples (83% [WT] < 89% [mut2]) (Appendix 1 Table 2). Although H-zWT was more sensitive than ZIKV WT in individual IgM/IgG tests, both antigens showed comparable combined sensitivity (Appendix 1 Table 2). The ZIKV WT, however, was more cross-reactive to DENV IgG (specificity 54% [H-zWT] < 71% [ZIKV WT] < 80% [H-zMut2]).

### Engineered NS1 Antigens for Rapid Test Assay

**Figure 4:**
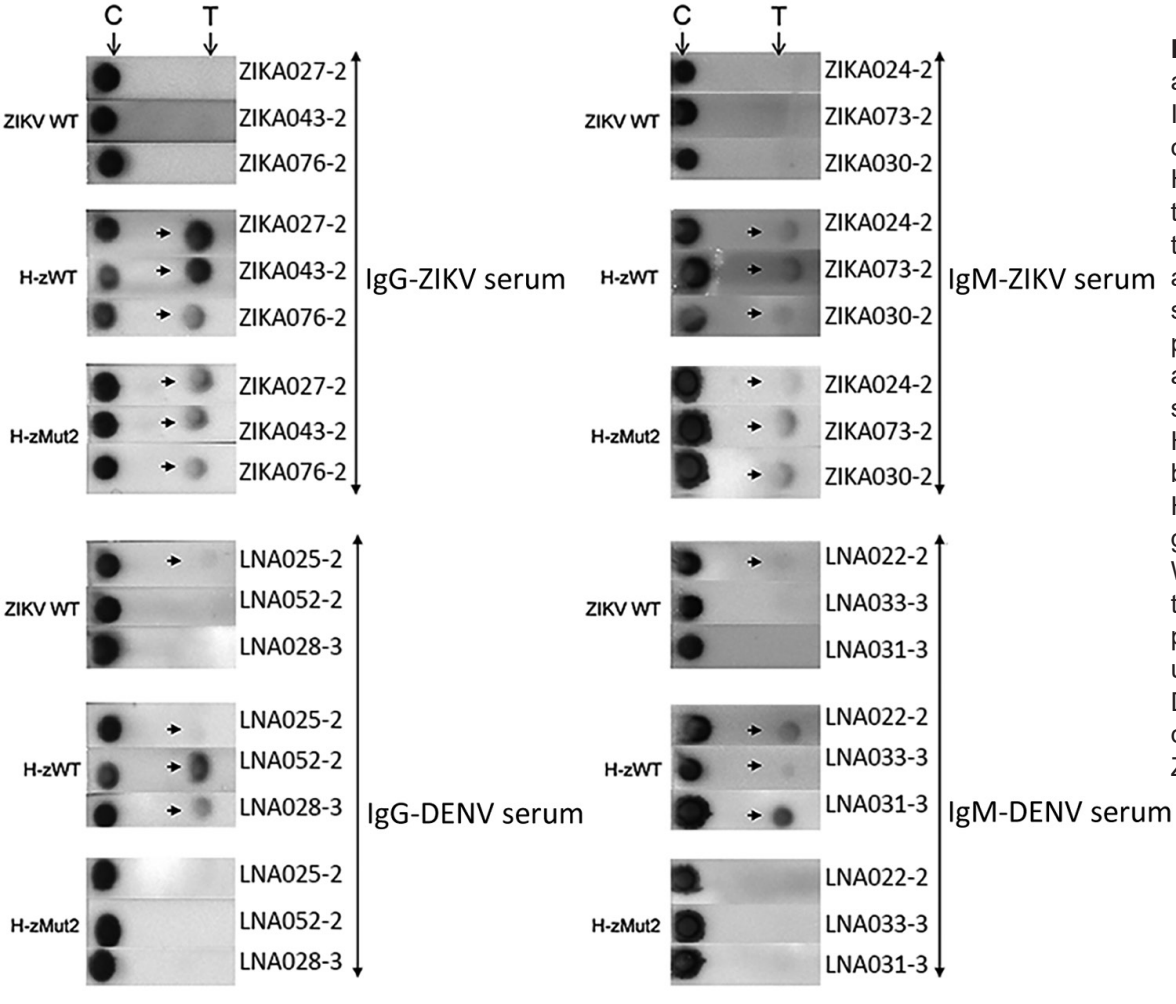
Immunochromatographic assay (IA) of H-zMut2 F1 IA for IgM and IgG detection in study of Zika diagnosis, Singapore. H-zMut2 as capture antigen in the F1 IA format was tested with training set for detecting IgG (left) and IgM (right). Representative strips show a comparison of performance for WT-NS1, H-zWT and H-zMut2. Overall, H-Mut2 showed higher specificity than H-zWT (against DENV plasma, bottom panels), though both H-Mut2 and H-zWT showed greater sensitivity compared to WT-NS1 (against ZIKV plasma, top panels). The arrows indicate positive signals at the test line (T), upstream of the control line (C). DENV, dengue virus; OD, optical density; WT, wild type; ZIKV, Zika virus.

**Figure 5:**
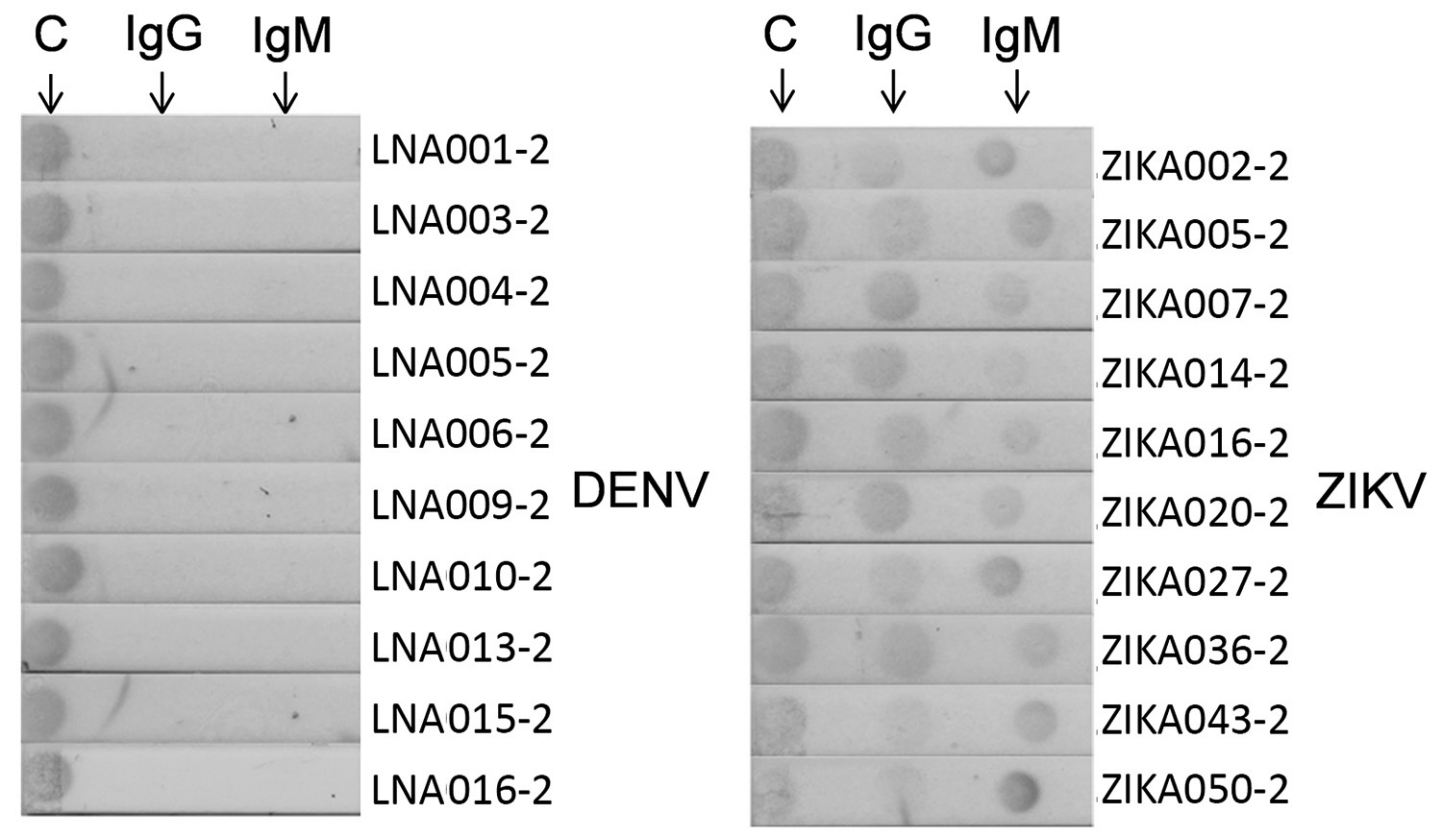
Immunochromatographic assay (IA) of H-zMut1 as detector antigen in the F2 IA for detecting IgM and IgG in study of Zika diagnosis, Singapore. Representative strips showing F2 IA format tested with validation set in blinded manner. Arrows at top indicate test lines. C, control line.

To develop IA that would permit rapid diagnosis of ZIKV infections, we evaluated both candidates, H-zMut1 and H-zMut2, using 2 different assay formats. The first format (F1), similar to the ELISA approach, used the engineered proteins as capture antigens for ZIKV IgM and IgG on 2 independent strips and used a detector antibody conjugated to enzyme for signal amplification (Figure 4). In the second format (F2), the antigens were conjugated to gold nanoparticles and served as a detector for binding patient IgM and IgG that were captured on 2 different spots on the same strip (Figure 5). During the development and optimization of the assays, we found that H-zMut2 showed better sensitivity than H-zMut1 in the F1 format, whereas HzMut1 showed better performance in the F2 format.

When analyzing the training set in the F1 format, H-zMut2 showed greater detection sensitivity and specificity than ZIKV WT (except slightly lower in IgM specificity, 89.6% [H-zMut2] vs. 95.5% [ZIKV WT]) and greater IgG specificity than H-zWT, though with comparable sensitivity (Figure 4; Appendix 1 Table 3). Although H-zWT also showed improved sensitivity compared with ZIKV WT (IgM 49% [WT] < 81% [H-zWT]; IgG 70% [WT] < 97% [H-zWT]), it showed lower IgG specificity than H-zMut2 and ZIKV WT (Figure 4; Appendix 1 Table 3).

### H-zMut2-F1 and H-zMut1-F2 for Blinded Test Evaluation

**Figure 6:**
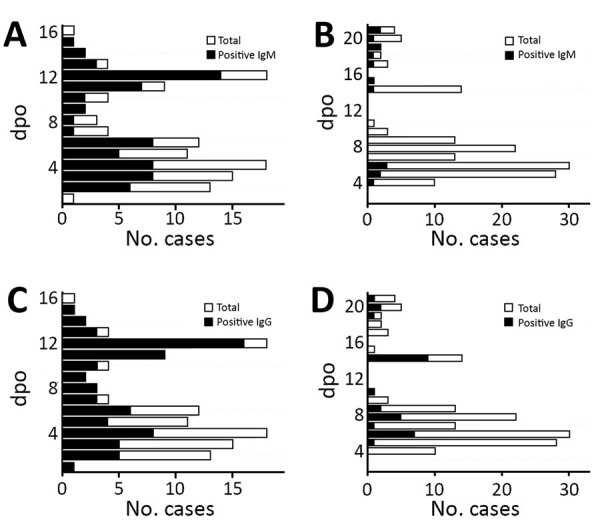
Distribution of number of plasma cases (*x*-axes) over number of DPO (*y*-axes) in study of Zika diagnosis, Singapore. F1 immunochromatographic assay format tested with validation set in a blinded manner (Tan Tock Seng Hospital plasma); positive plasma (black) and total plasma cases (gray) over dpo are also shown. A, C) Zika patient samples; B, D) Dengue patient samples. dpo, days postonset of symptoms.

When we evaluated the H-zMut2-F1 assay with the validation set in a blinded manner, it showed 51%/95% (IgM) and 44%/93% (IgG) sensitivity/specificity for the acute phase samples (Table 2; Figure 6). In contrast with the acute plasma samples, the F1 assay could achieve >70% test performance for convalescent samples (sensitivity: IgM/IgG 71%/90%; specificity: IgM/IgG 87%/79%). Combining both IgM and IgG tests increased the sensitivity for acute phase samples (69%) without greatly lowering the specificity (89% vs. 95%) (Table 2). Although the combined tests showed no major change in sensitivity with convalescent samples (90%), there was a slight decrease in the specificity (69% [IgM + IgG] <79% [IgG] <87% [IgM]) (Table 2).

When we used H-zMut1 in the F2 format to analyze the validation set, it showed lower sensitivity than HzMut2-F1, noticeably in IgG detection (Table 2). However, when both IgM and IgG tests were combined, H-zMut1-F2 showed improved sensitivity, 60% for acute samples and 88% for convalescent samples, while maintaining excellent specificity, 96% for acute samples and 84% for convalescent samples (Table 2).

### Performance Comparison for F1/F2 IA Format and Commercial Kit

We evaluated a commercially available ZIKV IgM/IgG rapid test kit (GenBody, http://genbody.co.kr) with TTSH samples, and compared the results to our F1 and F2 IA formats obtained from the blinded samples test. The GenBody kit used E (envelope) and NS1 antibodies in complex with E/NS1 antigen for detecting ZIKV IgM/IgG. This commercial kit was previously reported to exhibit high sensitivity and specificity for both IgM and IgG (>90%) (*24*). The GenBody tests did not perform as well as our F1 and F2 IA when applied to the samples from the validation set (Table 3). In particular, the Genbody test showed low sensitivity for IgM (29%) and low specificity for IgG (62%). The combined IgM/IgG test from GenBody showed low specificity (56%) but reasonable sensitivity (79%).

**Table 3:** Sensitivity and specificity comparison between GenBody and in-house IA assays for study of Zika diagnosis, Singapore*

Late phase, 7–16 dpo	Sensitivity, % (95% CI)				Specificity, % (95% CI)		
	GenBody	Lateral flow			GenBody	Lateral flow	
		F1	F2			F1	F2
IgM	28.6 (15.7–44.6)	76.2 (60.5–87.9)	73.8 (58.0–86.1)		97.4 (86.5–99.9)	100.0 (91.0–100.0)	94.9 (82.7–99.4)
IgG	71.4 (55.4–84.3)	85.7 (71.4–94.6)	76.2 (60.5–87.9)		61.5 (44.6–76.6)	79.5 (63.5–90.7)	89.7 (75.8–97.1)
IgM/IgG	78.6 (63.2–89.7)	85.7 (71.4–94.6)	85.7 (71.5–94.6)		56.0 (42.1–74.4)	79.5 (63.5–90.7)	84.6 (69.5–94.1)

*All IA assays were evaluated with TTSH plasma for IgM and IgG test (ZIKV, n = 42; DENV, n = 39, 7–16 dpo, subset of blinded test samples). GenBody strips were tested in a nonblinded approach, and compared with F1 and F2 results that were obtained from the blinded test of the validation set. DENV, dengue virus; dpo, days post onset of symptoms; F1, capture format; F2, detector format; IA, immunochromatographic assay; TTSH, Tan Tock Seng Hospital; ZIKV, Zika virus.

### Addition of ZIKV NS1 Test to Improve Sensitivity for Acute Phase Samples

Detecting DENV NS1 in serum has been reported to be a suitable method for diagnosing acute DENV infections (*25*,*26*). We hypothesized that by detecting NS1 antigen in acute ZIKV-infected plasma, this assay could improve the sensitivity of the IgM/IgG test because ZIKV belongs to the same flavivirus family as DENV. We generated monoclonal antibodies specific against ZIKV NS1 antigen and optimized antibody pairing for quantitative ELISA (Appendix 1 Figure, panel A). Using normal human serum spiked with recombinant ZIKV NS1, we established 0.1 ng/mL as the detection limit in our assay (Appendix 1 Figure, panel B). After testing 45 DENV samples, we set a cutoff above 0.25 ng/mL as being ZIKV NS1 positive (Appendix 1 Figure, panel C).

**Figure 7:**
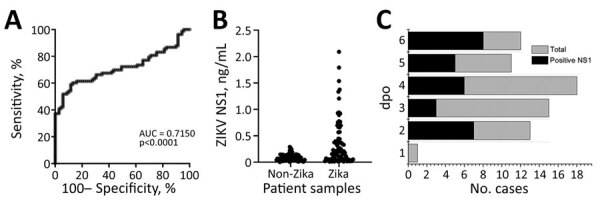
ELISA for ZIKV NS1 detection in study of Zika diagnosis, Singapore. A) Receiver operating characteristics curve analysis showing the performance of C12-C11 sandwich ELISA when tested against ZIKV-infected or non–ZIKV-infected samples. B) ZIKV NS1 quantification in patient samples using in-house antibody pairs. Each dot represents an individual patient sample. C) Distribution of number of plasma cases (*x*-axis) over dpo (*y*-axis) for ZIKV NS1 ELISA tested with the validation set; positive plasma (black) and the total plasma cases (gray) at each dpo are also shown. DENV, dengue virus; dpo, days postonset of symptoms; NS1, nonstructural protein 1; ZIKV, Zika virus.

We next evaluated the performance of our NS1 ELISA by testing the validation set in a blinded fashion. The area under the receiver operating characteristics curve plotted with ZIKV-infected and non–ZIKV-infected samples was 0.715, suggesting that the assay was able to differentiate between these 2 groups of patients with sensitivity of 41% and a specificity of 98% for acute phase samples (Table 2; Figure 7, panel A). We found that the ZIKV NS1 concentration was extremely low or undetectable in most of the patient samples. Among all the ZIKV-infected acute samples, only 7% had NS1 >1 ng/mL; 34% were in the range of 0.25–1 ng/mL, and 60% of the samples had NS1 level below the detection limit (Figure 7, panels B, C). However, when complementing NS1 antigen detection with either IgM or IgG ELISA, the sensitivity of detection could be improved for acute-phase infections (53% [IgM+IgG] < 56% [IgM+NS1] < 61% [IgG+NS1]) (Table 2). After we combined all 3 tests (NS1/IgM/IgG), the ELISA sensitivity was further improved to 67% while maintaining a high specificity (96%).

### Analysis of Acute-Phase Patient Samples

We tested a total of 151 acute-phase samples (70 ZIKV and 81 DENV samples, collected at 1–6 dpo) with ELISA and IA methods. Our data suggested that a combination of 3 immuoassays, NS1, IgM, and IgG, was needed to achieve a reasonable detection sensitivity in the acute phase. Among the 70 acute-phase serum samples, our ELISA tests were able to detect ZIKV infection as early as 2 days after fever onset, through detecting NS1 (7 cases), IgM (4 cases), or IgG (2 cases). The overall detection rate for the 70 acute-phase samples was 41% for NS1 (29 cases), 41% for IgM (29 cases), and 22% for IgG (16 cases). Only 8 of the 70 acute-phase samples were positive for both IgM and IgG. Among the 29 samples positive for NS1, 19 were positive for IgM and 2 were positive for IgG. Within the validation set (acute- and convalescent-phase samples, n = 118), 35 patients provided their blood samples at 2 different time points (Appendix 1 Table 4). We observed increased IgM and IgG levels in most of the samples by ELISA, upon disease progression over time (30 of 35 cases). For 28 of these patients, the first collection was in the acute phase and the second in the convalescent phase. We compiled test results and associated information for all patient samples used in this study (Appendix 2). 

## Discussion

In this study, we engineered ZIKV NS1 mutants for serologic testing in 2 different methods, the ELISA and the IA. We also developed monoclonal antibodies for detecting ZIKV NS1 to complement the serologic tests. A notable feature of our study was the ability to access confirmed ZIKV-infected and DENV-infected samples collected in acute and recent convalescent phases of infection (118 ZIKV samples, 151 DENV samples), which enabled a detailed evaluation and analysis of our assay’s performance.

The ZIKV IgM test was recommended by the Centers for Disease Control and Prevention (CDC) as part of the diagnostic regimen for symptomatic persons, as well as for nonsymptomatic pregnant women (*18*). The major drawback for serologic tests, including those authorized by CDC for emergency use, is the high rate of cross-reactivity to DENV-positive samples (*21*,*27*). A supplemental PRNT test is thus required to confirm IgM-positive specimens (*18*,*27*). Therefore, there is still a need for the development of a rapid, sensitive, and specific serologic test.

Both ZIKV E and NS1 antigens have been used in various serologic assays (*21*,*28*,*29*). In the ELISA format, both CDC and InBios (https://inbios.com) IgM kits used a monoclonal antibody that was previously developed against the West Nile virus E antigen. Although the 2 assays showed high positive test agreement (*21*), some studies demonstrated high false-positive rates with both assays (*21*,*30*). To reduce cross-reactivity to native DENV E antigen, either a mutated full-length or a conserved domain have been used (*31*,*32*). These capture antigen-based ELISA assays have some drawbacks, such as requiring a competing heterologous antigen to achieve better IgG specificity or showing cross-reactivity to recent convalescent-phase DENV samples obtained within 12 weeks of symptom onset. Good specificity was reported with the use of ZIKV NS1 as capture agent for serologic testing, but an evaluation study showed that the assays had low sensitivity (*29*).

The result of our blinded study indicated that the engineered H-zMut2 is suitable for developing a relatively reliable serologic ZIKV test, especially with convalescent samples (7–16 dpo). In comparison with the serologic assays reported by others, our ELISA tests showed reasonable performance characteristics for convalescent specimens and were relatively easy to perform. The entire assay can be completed within 90 min for IgM or 30 min for IgG, without the need to use a heterologous competing protein. Our tests also showed low cross-reactivity against recent convalescent-phase DENV samples (7–21 dpo).

We demonstrated the use of an engineered NS1 protein for accurate ZIKV diagnosis in both ELISA and IA approaches. The 2 IA formats were slightly different in test performance with convalescent samples. For example, the F1 IA approach showed favorable performance in individual tests (sensitivity/specificity 71%/87% for IgM, 90%/79% for IgG) whereas the F2 IA, albeit conferring lower individual test sensitivity (71% for IgM, 79% for IgG), had improved overall performance with >80% sensitivity and specificity in combined IgM/IgG tests. In addition, we found that our IA assays outperformed the GenBody RDT kit when tested against samples in the validation set.

On the basis of our ZIKV ELISA and IA test performance, we propose that patients being tested in the time window of 7–16 dpo can be evaluated by our IgM/IgG tests as part of the current diagnostic algorithm. These tests would potentially streamline the diagnostic process by reducing the dependency on PRNT. For patients in the acute phase, the combined NS1/IgM/IgG test would be appropriate. Even though the NS1 test by itself was not reliable for diagnosing early ZIKV infections, the inclusion of this test with IgM/IgG improved the overall sensitivity of the assay. Our specificity could be reduced when diagnosing acute ZIKV patients who might have had recent or remote DENV infections; a slight decrease in specificity was observed in the IgG test when comparing DENV convalescent and acute samples. These results indicate that NS1 alone is not sufficient for early diagnosis of ZIKV infection, in contrast to a report by Bosch et al. (*33*). The discrepancy could be the result of differences in the patients’ immune response or in the assay protocols.

In conclusion, we have developed a serologic test based on engineered NS1 mutants for detecting ZIKV IgM/IgG. Coupled with an NS1 antigen detection test, the combined NS1/IgM/IgG assay showed relatively high sensitivity and specificity and outperformed a commercial kit. Further evaluation using patient samples from different infected regions, ZIKV/DENV strains, and pandemic/epidemiologic records is needed to determine the overall performance of our assays. These assays, in either ELISA or IA format, can potentially be developed for on-site diagnosis to achieve better disease control and improved patient care during outbreaks of ZIKV infections.

Appendix 1 Additional information on detailed methods for the development of the Zika virus serological tests for Zika diagnosis, Singapore.

Appendix 2 Data from study on developing Zika virus serological tests for Zika diagnosis, Singapore.
