# Supplementary material for: Engineered NS1 for Sensitive, Specific Zika Virus Diagnosis from Patient Serology
Source: Emerg Infect Dis. 2021 May;27(5):1427–37. doi: 10.3201/eid2705.190121 (PMC8084482; doi:10.3201/eid2705.190121)
Supplement: Appendix 1 — Additional information on detailed methods for the development of the Zika virus serological tests for Zika diagnosis, Singapore. [file 19-0121-Techapp-s1.pdf]

Article DOI: https://doi.org/10.3201/eid2705.190121

Engineered NS1 for Sensitive, Specific Zika Virus Diagnosis from Patient Serology

Appendix 1

Detailed Methods for the Development of the Zika Serological Tests

Cloning, Expression, and Purification of Zika NS1 Antigens

Flaviviruses NS1 and ZIKV WT (WT-NS1, Uganda strain) were purchased from Native Antigen (https://thenativeantigencompany.com). Zika NS1 construct (French Polynesia/10087PF/2013; GenBank accession no. KX447521.1) was used for in-house protein engineering and expression. ZIKV NS1 mutants, peptide fragments, and domain fragments were generated from either gene synthesis (Integrated DNA Technologies [IDT], https://www.idtdna.com or Bio Basic, https://www.biobasic.com) or primers containing the designed residue mutations (IDT). Plasmids containing related ZIKV NS1 antibodies and ZIKV NS1 proteins were expressed in mammalian cells with ExpiCHO system (Thermo Fisher, https://www.thermofisher.com). Culture supernatants were harvested and purified using HisPur Ni-NTA resin (Thermo Fisher) for His-tagged proteins and protein A beads (Amintra) for antibodies. Proteins were buffer exchanged with PBS buffer (Thermo Fisher) using Amicon Ultra 15 (Millipore, https://www.sigmaaldrich.com), and quantified by using a NanoDrop spectrophotometer (Thermo Fisher).

Immunochromatographic Assays

Format 1: Nitrocellulose membrane strips (Millipore) were spotted with 0.33 μL of 0.5 mg/mL H-zMut2 (test line) and 0.33 μL of 0.2 mg/mL polyclonal goat anti-human IgG or 0.33 μL of 0.2 mg/mL mouse anti-human IgM antibody (control line). The strips were dried at 37°C for 5 min and blocked with blocker casein in TBS for 30 min. The strips were then washed with borate buffer supplemented with 1% sucrose and 0.01% SDS and dried for 1 hour at 37°C. The strips were assembled with the glass fiber filter and absorption pad. For the IgM test, 5 μL of diluted sample (1:220) was passed through the membrane, followed by 15 μL of PBS buffer

containing 0.05% Tween and 1% BSA. The strips were then added to polyclonal goat anti-human IgM-AP (Fitzgerald, https://www.fitzgerald-fii.com), followed by 10 μL of washing buffer. Subsequently the strip was dipped in 200 μL of BCIP/NBT (MOSS) for 7.5 min. The reaction was stopped by dipping the membrane strip in 0.3 M NaOH. For the IgG test, 5 μL of diluted sample (1:400) was passed through the membrane, followed by 15 μL of the washing buffer. The strips were then added to goat anti-human IgG Fc HRP (Thermo Fisher), followed by 10 μL washing buffer. The membrane strip was dipped in 200 μL of metal-enhanced DAB solution (Thermo Scientific) for 5 min. The strips were dipped in water before imaging. All the strips were imaged using a Bio-Rad ChemiDoc MP Imaging System (https://www.bio-rad.com) on autoscale setting.

Format 2: To prepare the test strips, 1 μL of anti-human IgG and IgM capture antibodies (1 mg/mL) were immobilized on nitrocellulose membrane strips (3 mm width × 25 mm length) at the downstream and upstream portion, respectively, via vacuum drying. The test strips were then blocked with casein (1% w/v), washed with borate buffer, and vacuum dried before use. To prepare the conjugate pad, the H-zMut1 antigen was first conjugated to 40-nm gold nanoparticles (Au NPs) via covalent binding. The conjugated Au NPs were then diluted to 0.5 optical density (OD) using casein buffer, and then dried on glass fiber strips (3 mm width × 30 mm length). The nitrocellulose test strips were assembled with the glass fiber and an absorbent pad (10 mm width × 20 mm length). Patient plasma samples (neat, 5 μL) were applied to the upstream portion of the nitrocellulose test strip and 60 μL of chasing buffer (1× PBS) was then applied to the glass fiber conjugate pad. As the patient plasma and Au NPs flowed past the IgG/IgM test spots, a visible red signal could be observed by the naked eye within 15 minutes.

ZIKV IgM and IgG ELISA Assay

Polystyrene plates were coated overnight with 1 μg/mL of ZIKV NS1-related antigen in PBS buffer, and blocked with blocking buffer (PBS with 10% nonfat dry milk [Bio-Rad]). To perform the ZIKV IgM ELISA assay, patient serum diluted in blocking buffer was mixed with IgG/Rf stripper (Bio-Rad) (e.g., 0.5 μL of sample with 2 μL of IgG stripper in a total of 60 μL to make a 1:120 sample dilution) and incubated for 30–45 min. After incubation, the sample was transferred to the ZIKV NS1-coated plates and incubated for 25 min at 37°C. Plates were washed, and anti-human IgM-HRP (Abcam, https://www.abcam.com) (1:4300 dilution) was

added for an additional 10 min incubation at 37°C. Plates were washed again, and the TMB substrate was added for 10 min before stopping with KPL stop solution (SeraCare, https://www.seracare.com). For the ZIKV IgG ELISA assay, blocking buffer containing patient serum (1:250 dilution) was transferred to ZIKV NS1 antigen-coated plates and incubated for 12 min at 37°C. Upon washing, anti-human IgG-HRP (Thermo Fisher) (1:5500 dilution) was added to the plates for 10 min incubation at room temperature, followed by substrate incubation for 7 min, and stopped by adding stop solution and measuring absorbance at 450 nm. Serum samples' mean ODs were measured from 2 replicates. For the ELISA assays performed using serum samples from the training and validation sets, mean OD of the sera samples (P) was divided by the mean OD of an internal standard (I). P/I ratio of >1.5 was considered as Zika positive for both IgM and IgG assays. The internal standard was built based on a commercial dengue sample that consistently showed minimal cross reactivity in our assays.

Monoclonal Antibody Generation and Production

The anti-ZIKV NS1 antibodies were generated in New Zealand white rabbits according to an approved Institutional Animal Care and Use Committee protocol. Briefly, each animal was immunized with recombinant ZIKV WT NS1 (Native Antigen) and the titer was checked after every round of immunization. Upon achieving a high titer, B cells were isolated and sorted for culture. Supernatants from the B cells were tested using ZIKV NS1 antigen ELISA and variable regions were then recovered from positive clones for subcloning into expression vector containing rabbit constant regions. Monoclonal antibodies were expressed in CHO cells for large-scale production according to manufacturer's protocol (Gibco, https://www.thermofisher.com). Antibodies were purified with Protein A resins (Amintra; Abcam), buffer-exchanged into PBS (Gibco), and concentrated using Amicon Ultra centrifugal filters (Merck Millipore).

ZIKV NS1 ELISA

Polystyrene plates were coated with 1 μg/mL of C12 in PBS and incubated overnight at 4°C. The plates were blocked with 2% bovine serum albumin, fraction V (BSA, Capricorn, https://www.capricorn-scientific.com) in PBS before use. After washing with PBS, 45 μL of serially diluted recombinant ZIKV NS1 antigen (ranging from 0 to 6.4 ng/mL) in normal human serum control or patient sample were co-incubated with 5 μL of 10% BSA and 1% PBST for 1

hr at 37°C; 10 µg/mL of recombinant DENV1 NS1 in normal human serum control was included as negative control. Plates were then washed 5 times with 0.2% PBST and once with PBS. Next, an optimized amount of biotinylated C11 in 1% BSA 0.1% PBST was added for a 1-hour incubation at room temperature. The plates were washed and incubated at room temperature with streptavidin-poly-HRP (SDT) diluted in 1% BSA 0.1% PBST for 30 min. The plates were then developed with 100 µL of TMB for 15 min and terminated with 50 µL of stop solution. The absorbance at 450 nm was taken using a Tecan M200 plate reader (Thermo Fisher). The limit of detection was set at 2× the OD_{450} of the background (normal human serum). The ZIKV NS1 level in the samples was estimated through interpolation from a standard curve. The cutoff was established by 3 standard deviations (SD) from the mean values of 45 DENV patient samples interpolated from the standard curve.

Appendix 1 Table 1. H-zMut2 ELISA for training set*

No. samples tested	ZIKV case sensitivity, % (positive cases detected/total)	DENV case specificity, % (not detected/total)
No. samples tested, IgM test	37<br>83.4 (31/37)	52<br>86.5 (45/52)
No. samples tested, IgG test	37<br>89.2 (33/37)	67<br>88.1 (59/67)

*Both IgM and IgG ELISA were tested with plasma as indicated by using H-zMut2 as the capture antigen. Details of the training set were described in the Materials and Methods section (main article). For the ZIKV IgM test, 27 ZIKV plasma and 31 DENV plasma samples were obtained from TTSH, and 10 ZIKV sera and 21 DENV sera were obtained from commercially available sources. For the ZIKV IgG test, 27 ZIKV plasma and 46 DENV plasma samples were obtained from TTSH, and 10 ZIKV serum samples and 21 DENV serum samples were obtained from commercially available sources. DENV, dengue virus; ELISA, enzyme-linked immunosorbent assay; TTSH, Tan Tock Seng Hospital; ZIKV, Zika virus.

Appendix 1 Table 2. Capture antigens-ELISA for validation set*

Antigen	ZIKV case sensitivity, % (positive cases detected/total)		DENV case specificity, % (not detected/total)	
	Acute	Convalescence	Acute	Convalescence
Antigen: H-zMut2				
IgM test	41.4 (29/70)	79.2 (38/78)	100.0 (81/81)	95.7 (67/70)
IgG test	22.9 (16/70)	83.3 (40/48)	97.5 (79/81)	84.3 (59/70)
Combined IgM and IgG test	52.9 (37/70)	89.6 (43/48)	98.8 (80/81)	80.0 (56/70)
Antigen: ZIKV WT				
IgM test	2.9 (2/70)	33.3 (38/48)	97.5 (79/81)	98.6 (69/70)
IgG test	14.3 (10/70)	56.3 (27/48)	97.5 (79/81)	72.9 (51/70)
Combined IgM and IgG test	17.1 (12/70)	83.3 (40/48)	97.5 (79/81)	71.4 (50/70)
Antigen: H-zWT				
IgM test	51.4 (36/70)	81.3 (39/48)	95.1 (77/81)	74.3 (52/70)
IgG test	30.0 (21/70)	85.4 (41/48)	96.3 (78/81)	68.6 (48/70)
Combined IgM and IgG test	65.7 (46/70)	90.0 (43/48)	92.6 (72/81)	54.3 (38/70)

*The capture antigens**,** H-zMut2, WT-NS1, and H-zWT, were tested for detecting IgM and IgG in a blinded manner; sensitivity and specificity were determined against ZIKV and DENV plasma, respectively. In the combined IgM/IgG tests, normalized OD >1.5 either in IgM or IgG test for respective plasma sample was determined as positive. Plasma sample details and information were described in the Material and Methods section in the main article. DENV, dengue virus; OD, optical density; ZIKV, Zika virus.

.

Appendix 1 Table 3. Capture antigens in immunochromatographic assay (F1 IA) for IgM and IgG detection*

Category	ZIKV cases sensitivity, % (positive cases detected/total)	DENV case specificity, % (not detected/total)
No. samples tested	37	67
Antigen: H-zMut2		
IgM test	89.2 (33/37)	89.6 (60/67)
IgG test	97.3 (36/37)	94.0 (63/67)
Antigen: ZIKV WT		
IgM test	48.6 (18/37)	95.5 (64/67)
IgG test	70.3 (26/37)	74.6 (60/67)
Antigen: H-zWT		
IgM test	81.1 (30/37)	83.6 (56/67)
IgG test	97.3 (36/37)	59.7 (40/67)

*The capture antigens, H-zMut2, WT-NS1 and H-zWT, were tested with the training set as defined in the Materials and Methods section (main article). Both IgM and IgG detection in the F1 IA format were evaluated with samples as indicated. For ZIKV IgM and IgG tests, 27 ZIKV plasma samples and 46 DENV plasma samples were obtained from TTSH, and 10 ZIKV serum samples and 21 DENV serum samples were obtained from commercially available sources. DENV, dengue virus; TTSH, Tan Tock Seng Hospital; ZIKV, Zika virus.

Appendix 1 Table 4. H-zMut2 ELISA for patient plasma samples collected over 2 time points

Sample no.	dpo	Normalized IgG	Normalized IgM		dpo	Normalized IgG	Normalized IgM
ZIKA002-1	6	0.245056	7.009544		7	0.536954	12.77939
ZIKA005-1	3	0.2292	1.072633		12	6.523791	11.7708
ZIKA007-R	2	0.160063	0.944636		3	0.252408	1.886678
ZIKA024-R	8	7.055933	1.110142		10	11.63988	1.299192
ZIKA014-R	6	0.148191	0.432131		7	0.390206	0.454905
ZIKA015-R	5	0.112273	0.976121		6	0.118206	14.78041
ZIKA016-1	3	0.248569	1.151707		12	7.452582	13.3077
ZIKA018-1	5	0.166857	4.758398		14	3.166973	11.52558
ZIKA020-1	5	3.009509	2.456247		12	7.836708	8.926948
ZIKA024-1	7	4.96869	0.798065		8	7.055933	1.110142
ZIKA027-1	3	0.17766	5.043171		12	6.397515	16.96656
ZIKA028-1	4	1.081659	0.548624		11	8.647051	1.993418
ZIKA029-1	5	0.112195	0.764514		12	0.173767	0.798875
ZIKA030-1	4	1.876883	0.917834		12	11.48693	1.762583
ZIKA034-R	4	1.481061	0.898225		12	5.706225	1.045856
ZIKA036-1	5	3.119371	3.789137		13	6.898693	7.675395
ZIKA043-R	2	0.506782	1.909105		4	1.843062	7.526417
ZIKA050-1	6	0.566019	2.051754		12	7.508097	21.44605
ZIKA053-1	6	0.421136	4.078114		12	4.492642	15.71716
ZIKA054-1	5	0.774091	0.542287		6	2.144467	0.959967
ZIKA055-1	6	0.196445	1.288362		11	1.383456	12.63373
ZIKA056-1	6	0.223802	1.698473		12	3.418552	21.10136
ZIKA060-R	4	0.357674	1.549509		9	5.941243	22.28346
ZIKA061-1	4	0.134477	0.391196		5	0.147088	0.488138
ZIKA067-R	4	1.210687	0.613105		5	3.095906	1.017795
ZIKA068-1	4	0.227564	0.325781		10	0.178818	0.50098
ZIKA069-R	4	6.244419	0.415801		5	7.062833	0.399823
ZIKA070-1	3	0.338197	0.432599		4	0.283432	0.45979
ZIKA072-1	4	2.519165	0.445334		12	10.70816	7.508905
ZIKA073-1	4	0.390882	8.400598		12	6.837443	8.630448
ZIKA075-1	3	5.60034	2.777571		11	8.339426	25.36846
ZIKA076-1	2	0.152257	0.681845		12	5.555499	19.51593
ZIKA079-1	4	2.275925	1.932228		12	7.498631	10.29619
ZIKA083-1	6	0.531495	0.442915		12	0.457126	1.024719
ZIKA088-1	7	6.968271	5.710357		13	7.187742	11.81088

*A total of 35 patient specimens were assayed for detecting ZIKV IgM and IgG. Increase in the normalized OD for both IgM and IgG can be observed in most cases at the second time point, upon disease progression (30/35 cases); 28 of the 35 patients were tracked from acute to convalescent phase (n = 28). Normalized OD >1.5 was highlighted in gray. dpo, days post onset of symptoms; OD, optical diameter.

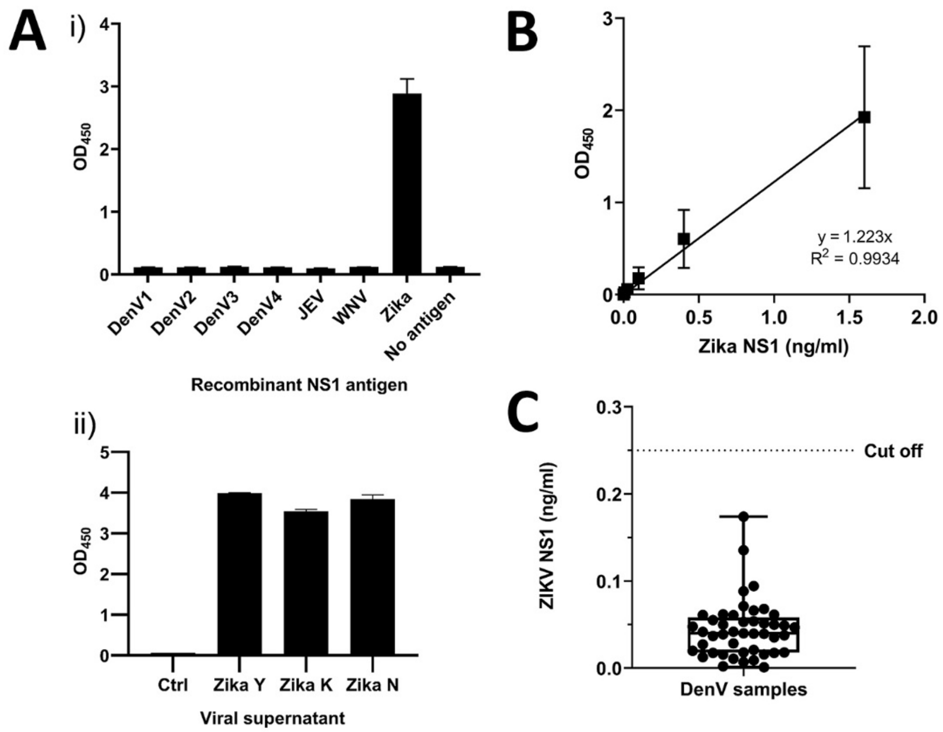

Appendix 1 Figure. ZIKV NS1 “sandwich” ELISA using in-house antibody pair. A) C12-C11 was tested by ELISA using recombinant antigens of different flaviviruses spiked into human control serum (panel i) and viral culture supernatant (panel ii). Zika Y, Puerto Rico strain (Y2015); Zika K, Thailand strain (KF993678); Zika N, Singapore strain (NPHL). The data represent the average (±SD) of the replicates for each sample from 1 of the 3 independent experiments. B) Standard curve for ZIKV NS1 ELISA established with recombinant protein spiked into human serum. The assay values represent the average of 4 experiments, and the error bars indicate the SD. C) The cutoff for ELISA was established using 45 patient samples infected by DENV. Horizontal line indicates cutoff at 0.25 ng/mL. DENV, dengue virus; ELISA, enzyme-linked immunosorbent assay; SD, standard deviation; ZIKV, Zika virus.
